# A survey of *Methylobacterium* species and strains reveals widespread production and varying profiles of cytokinin phytohormones

**DOI:** 10.1186/s12866-022-02454-9

**Published:** 2022-02-08

**Authors:** Daniel Palberg, Anna Kisiała, Gabriel Lemes Jorge, R. J. Neil Emery

**Affiliations:** 1 Environmental and Life Sciences Graduate Program, Trent University, 1600 West Bank Drive, Peterborough, ON K9L 0G2 Canada; 2 Department of Biology, Trent University, 1600 West Bank Drive, Peterborough, ON K9L 0G2 Canada; 3 Department of Technology, Sao Paulo State University, Jaboticabal, Sao Paulo, Brazil

**Keywords:** 2-methylthio-Zeatin, HPLC–MS/MS, Methanol, Plant growth promoting bacteria, trans-Zeatin

## Abstract

**Background:**

Symbiotic *Methylobacterium* strains comprise a significant part of plant microbiomes. Their presence enhances plant productivity and stress resistance, prompting classification of these strains as plant growth-promoting bacteria (PGPB). *Methylobacteria* can synthesize unusually high levels of plant hormones, called cytokinins (CKs), including the most active form, trans-Zeatin (tZ).

**Results:**

This study provides a comprehensive inventory of 46 representatives of *Methylobacterium* genus with respect to phytohormone production in vitro, including 16 CK forms, abscisic acid (ABA) and indole-3-acetic acid (IAA). High performance-liquid chromatography—tandem mass spectrometry (HPLC–MS/MS) analyses revealed varying abilities of *Methylobacterium* strains to secrete phytohormones that ranged from 5.09 to 191.47 pmol mL^−1^ for total CKs, and 0.46 to 82.16 pmol mL^−1^ for tZ. Results indicate that reduced methanol availability, the sole carbon source for bacteria in the medium, stimulates CK secretion by *Methylobacterium*. Additionally, select strains were able to transform L-tryptophan into IAA while no ABA production was detected.

**Conclusions:**

To better understand features of CKs in plants, this study uncovers CK profiles of *Methylobacterium* that are instrumental in microbe selection for effective biofertilizer formulations.

**Supplementary Information:**

The online version contains supplementary material available at 10.1186/s12866-022-02454-9.

## Background

Bacteria which colonize plants inhabiting the root zone (rhizospheric), leaf surfaces (epiphytic), and living within tissues (endophytic), are numerous and diverse [1]. These plant-associated microbes play critical roles in plant health, development, and [2–7]. A better understanding of the biochemical bases of the interactions between plants and beneficial microbes is critical for improving plant performance in a sustainable manner [6, 7].

The *Methylobacterium* genus often comprises a significant part of the plant microbiome (phytobiome). Generally, *Methylobacteria* are ubiquitous in nature and non-pathogenic to humans or wildlife. They are rod-shaped, obligately aerobic microbes that can thrive in a wide range of environments including soil, air, water, and plants [8]. Most *Methylobacterium* species stain gram-negative and exhibit polar growth, although some exceptions exist (e.g., *M. jeotgali*) [9]. The distinct pink pigmentation of many *Methylobacterium* strains indicates the presence of specific carotenoids which may confer their tolerance to ultraviolet (UV) radiation [10–12] and provide a basis for further classifying individuals as pink-pigmented facultative methylotrophs (PPFMs) [1, 13, 14]. Pink pigmented facultative methylotrophs have been suspected to participate in the development of pigmentation within their host-plants by modulating flavonoid and carotenoid levels within host tissues [15]. As facultative methylotrophs, *Methylobacteria* can either use common carbon sources such as carbohydrates, or oxidize a range of single-carbon compounds including methanol, methylamine, and formaldehyde [1, 14, 16–18].

*Methylobacterium* strains can thrive across a wide range of temperatures, salinity, and pH [9], with certain strains exhibiting considerable tolerance to chlorine [19] and gamma irradiation [20]. *Methylobacterium* have been studied for their application in several technologies including bioremediation of environmental toxins [21, 22]. Most recently, novel strains of *Methylobacterium* were isolated from the international space station (ISS); seeming to have evaded decontamination measures and proving suitability for an oligotrophic environment in microgravity [23].

Early studies showed that enrichment of the phytobiome with *Methylobacterium* encouraged plant growth and productivity [24, 25], leading to the classification of these strains as plant growth-promoting bacteria (PGPB). These first series of discoveries led to the hypothesis that *Methylobacterium* may be a major source of plant growth hormones known as the cytokinins (CKs) [24]. Cytokinins are N^6^ substituted adenine derivatives, such as the zeatins (isoprenoid functionality) and topolins (aromatic functionality). Cytokinins are involved in a wide array of biological functions in plants including cell division and elongation, shoot growth, nutrient uptake, vascular development, and gametophyte development [26–28]. It has been proposed that CKs are produced by *Methylobacterium* as downstream products of tRNA degradation [29].

A unique trait of *Methylobacterium* genus that distinguishes it from other PGPB is their ability to biosynthesize unusually high levels of the most active CK forms including trans-Zeatin (tZ). By producing bioactive CKs that are identical in chemical structure and bioactivity to those endogenously produced by plants, symbiotic *Methylobacterium* can stimulate plant cell division and increase the release of methanol, a by-product of cell wall construction [18, 25, 30]. Thus, while the plant is stimulated to grow, *Methylobacterium* can receive and utilize single-carbon compounds like methanol as a stable carbon source [31]. In this interaction between plant and symbiont, *Methylobacterium* have a clear advantage as many other microorganisms in the phytobiome have more complex habitat requirements and rely on C_6_ nutrient sources that are subject to higher competition [17]. This attribute may prove especially advantageous for *Methylobacteria* in regions with a challenging climate and low soil fertility [32], or environments with limited resource bandwidth and renewability like space farming.

The production of growth-promoting phytohormones by *Methylobacterium* has generated interest in using these strains as bioinoculants in sustainable crop production [6]. Studies have illustrated how enrichment of a plant microbiome with *Methylobacterium* increases growth rate, as well as plant tolerance to high salinity [33] or drought stress [34]. The use of microbes like *Methylobacterium* to support growth and development of crops would be more technically efficient and cost-effective compared to genetic engineering procedures. Moreover, the microbial delivery of growth-promoting phytohormones would be more widely accepted by public consumers relative to more controversial GMO approaches.

The development of a successful bioinoculant begins with the selection of a suitable microbial agent for the target host-plant. Thus far, however, available studies examining CK biosynthesis in *Methylobacterium* are limited in both the number of strains evaluated and the range of CK forms analyzed. Nevertheless, existing research indicates that understanding the types and quantities of the CK forms produced by *Methylobacterium* can help elucidate how selected strains facilitate a beneficial effect on the host-plant and provide a foundation for novel and sustainable crop production technologies [35, 36].

In this study, we expand the knowledge of PGPB potential by producing a comprehensive inventory of 46 *Methylobacterium* strains with respect to their ability to produce and release phytohormones during in vitro culture. The analysed strains were specifically selected to capture a wide range of biometric and phenotypic traits, as well as different sources of origin. Microbial profiles of CK phytohormones, as well as their precursors and derivatives were analysed using the highly sensitive and accurate method of high-performance liquid chromatography—positive electrospray ionization—tandem mass spectrometry (HPLC-(+ ESI)-MS/MS). Furthermore, the analysis of CK secretion by the plant growth promoting strain, *M. oryzae* LMG23582(T), indicated that the hormone biosynthesis is more intense in the media depleted of methanol. To add further phytohormonal context, the bacterial strains were analyzed regarding production of a common CK antagonist, abscisic acid (ABA) through mass spectrometry, and a frequent complementary growth promoting compound, indole-3-acetic acid (IAA) through a colorimetric method.

## Results

### Cytokinins (CKs) and abscisic acid (ABA) profiling in *Methylobacterium* strains

The collection of 46 *Methylobacterium* strains analysed in this study represented a wide range of growth habitats. Most of the strains originated from plant organs and soil while other strains were derived from water, air and other sources (Table 1). All bacterial isolates were cultured in a minimum medium supplemented with methanol as a sole carbon source (DSMZ-125). Use of a minimum medium was essential to ensure that CKs detected using HPLC–MS/MS were strictly of bacterial origin and not background signal contribution from the constituents of nutrient-rich growth media [37].

**Table 1:** Inventory of *Methylobacterium* strains evaluated for phytohormone production

Species	Strain	Isolation Source	Characteristics/Application (as per depository info)
*M. aerolatum*	JCM 16406(T)	air	None Specified
*M. aminovorans*	LMG 21752(T)	soil	degrades tetramethylammonium hydroxide
*M. aquaticum*	B-59286	Phoenix spacecraft surface	None Specified
	DSM 23931	*Arabidopsis thaliana* — silique surface	None Specified
*M. bullatum*	LMG 24788(T)	greenhouse, cord moss (*Funaria hygrometrica*) — phyllosphere	None Specified
*M. cerastii*	DSM 23679(T)	*Cerasium holosteoides* — phyllosphere	None Specified
*M. extorquens*	B-1048	garden soil enriched with sarcosine	taxonomy, transformation host; utilizes alkylamine, formate, oxalate, methanol, methylamine; facultative methylotroph
	JCM 2805	air	taxonomy, genome sequenced strain; facultative methylotroph; used in studies of C1 metabolism; utilizes methanol, methylamine
	NBRC 15687(T)	soil	sterility assurance (antibiotic resistant); utilizes methanol, methylamine, oxalate; facultative methylotroph
	JCM 2806	garden soil, slough	utilizes methanol (methanol oxidaze), methylamine; facultative methylotroph
	DSM 13060	pine (*Pinus sylvestris*) meristem tissue cultures	None Specified
	DSM 23939	*Arabidopsis thaliana* — phyllosphere	None Specified
	JCM 2803	*Psychotria mucronata* — surface sterilized leaf nodules	None Specified
	JCM 20693	mine water	None Specified
*M. extorquens*	NBRC 103126	soil-litter close to *Rumex* sp.	Degrades oxalate
	NBRC 103127	soil-litter close to *Arum* sp.	Degrades oxalate
	NBRC 103129	soil-litter close to *Eucalyptus* sp.	Degrades oxalate
*M. gnaphalii*	NBRC 107716(T)	*Gnaphalium spicatum*	None Specified
*M. goesingense*	DSM 21331(T)	*Thlapi goesingense* — endosphere of the Ni-hyperaccumulating plant	resistance to Ni, Cd and Zn
*M. gossipiicola*	B-51692 (T)	cotton (*Gossipium hirsutum*) — phyllosphere	produces auxin and ACC deaminase
*M. iners*	JCM 16407(T)	air	None Specified
*M. jeotgali*	LMG-23639(T)	traditional fermented seafood (jeotgal)	None Specified
*M. marchantiae*	DSM 21328(T)	liverwort (*Marchantia polymorpha*) - phyllosphere (thallus)	None Specified
*M. mesophilicum*	B-14246 (T)	perennial rye grass (*Lolium perenne*) – healthy, green leaves	utilizes methanol, methylamine; facultative methylotroph; antibiotic resistant
	B-2390	household well water	None Specified
*M. nodulans*	LMG-21967(T)	*Crotalaria podocarpa* — nodules	induces nitrogen-fixing root nodules on legume Crotalaria spp.
*M. organophilum*	LMG-6083(T)	lake water, lake sediment	genetic transformation; enzyme regulation; facultative methylotroph; utilizes methanol, methylamine, trimethylamine; does not utilize methane; produce poly(3-hydroxybutyrate-co-3-hydroxyvalerate
*M. organophilum*	NBRC 103120	*Ficus elastica* — petiole maceration	degrades oxalate
	NBRC 103121	*Begonia* sp. — petiole maceration	degrades oxalate
*M. oryzae*	LMG-23582(T)	*Oryzae sativa* cv Nam-Pyeoung — surface-disinfected stem	possesses ACC deaminase activity; produces cytokinins
*M. oxalidis*	NBRC 107715(T)	*Oxalis corniculata*	None Specified
*M. phyllosphaerae*	LMG-24361(T)	*Oryza sativa* cv. Dong-Jin — leaf tissues	possesses ACC deaminase activity
*M. platani*	JCM 14648(T)	*Platanus orientalis* — leaf	None Specified
*M. radiotolerans*	LMG-2269(T)	Japanese unpolished (unhulled) old and commercial rice grain	radiation resistant; utilizes methanol, methylamine; facultative methylotroph; produces pigment (alpha-bacterioruberin)
	LMG-6379	forest soil	utilizes methanol, methylamine; facultative methylotroph; antibiotic resistant
*M. rhodinum*	LMG-2275(T)	Alder (*Alnus*) rhizosphere	utilizes methanol, methylamine; facultative methylotroph
*M. thiocyanatum*	JCM 10893(T)	*Allium aflatuense* – rhizosphere soil	degrades cyanate and thiocyanate
	NBRC 103122	*Bryophyllum* sp. — petiole maceration	degrades oxalate
	NBRC 103124	*Mesenbryanthemum* sp. — stem maceration	degrades oxalate
	NBRC 103128	soil-litter close to *Mesenbryanthemum* sp.	degrades oxalate
	NBRC 103130	soil-litter close to *Rumex* sp.	degrades oxalate
*M. zatmanii*	LMG-6087(T)	fermentor operating with formaldehyde as sole source of carbon	utilizes methanol, methylamine, trimethylamine facultative methylotroph
*Methylobacterium* spp.	LMG-6378	red discolouration of ginned cotton lint	utilizes methanol, methylamine; facultative methylotroph; antibiotic resistant
	DSM 23935	*Cardamine hirsuta* — phyllosphere	None Specified
	DSM 23936	*Medicago truncatula* — phyllosphere	None Specified
	JCM 14673	*Oryza sativa* SC-41 — stem of cultivated rice	None Specified
	JCM 14674	*Oryza rufipogon* W1964 — stem of wild rice	None Specified

**Fig. 1:**
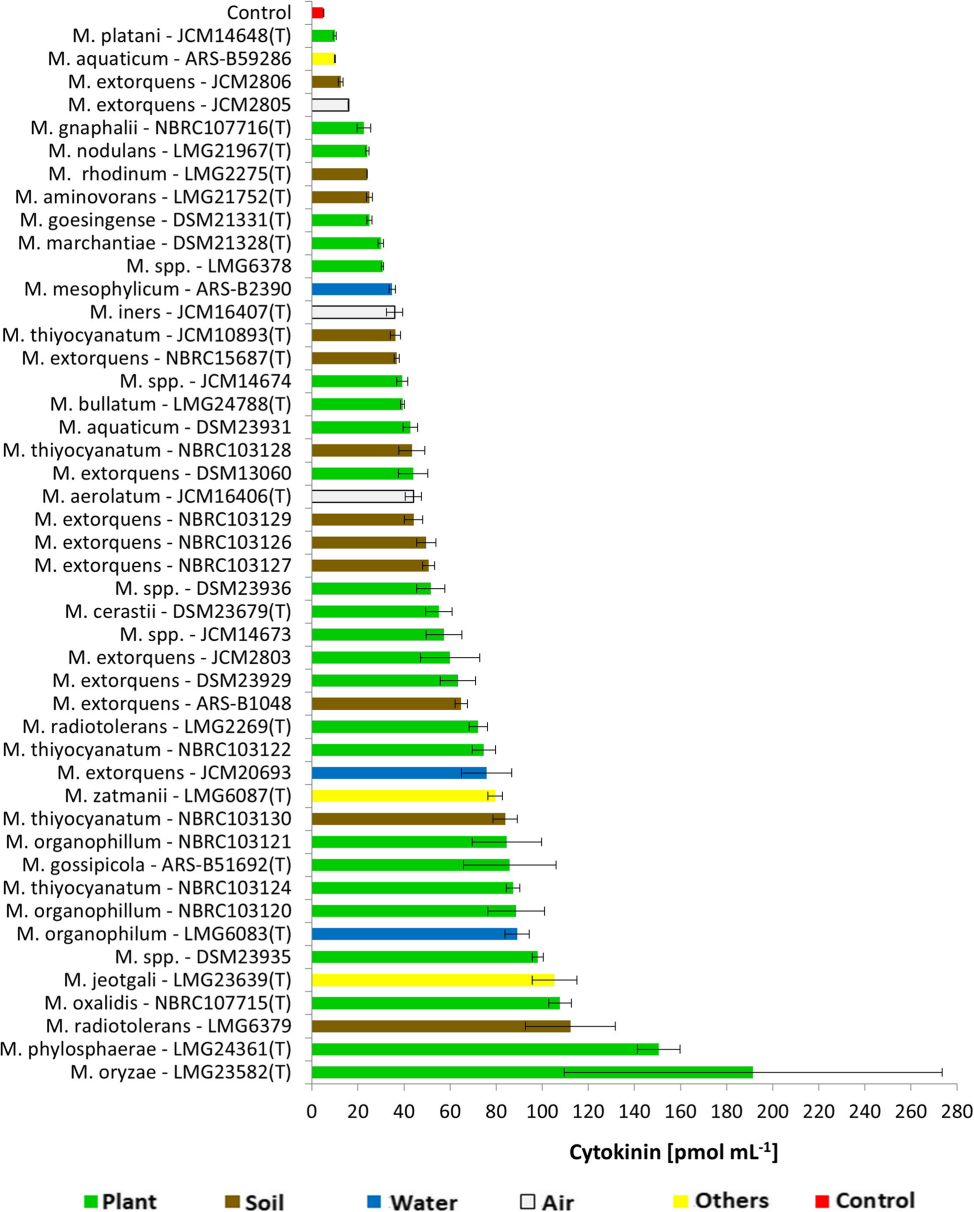
Total cytokinin (CK) concentration (pmol mL^−1^) in 46 *Methylobacterium* strains. Strains were cultured in vitro in the DSM125 minimal medium until they reached the early stationary phase. Cytokinin were analysed in the cell-free bacteria supernatants using HPLC-(ESI +)MS/MS. Values are means ± SE of 3 replicates

Total CK concentration in the cell-free supernatants of *Methylobacterium* strains ranged from 9.9 (*M. platani* JCM14648) to 191.5 pmol mL^−1^ (*M. oryzae* LMG23582) (Fig. 1). Regarding the total CK levels secreted by bacteria, out of the 5 most productive strains (over 100 pmol mL^−1^ CKs), 3 were isolated from plant organs (*M. oryzae* LMG23582(T), *M. phylosphaerae* LMG24361(T), and *M. oxalidis* NBRC107715(T)), while the remaining two (*M. radiotolerans* LMG6379 and *M. jeotgali* LMG23639(T)), originated from the forest soil and the fermented food, respectively.

**Fig. 2:**
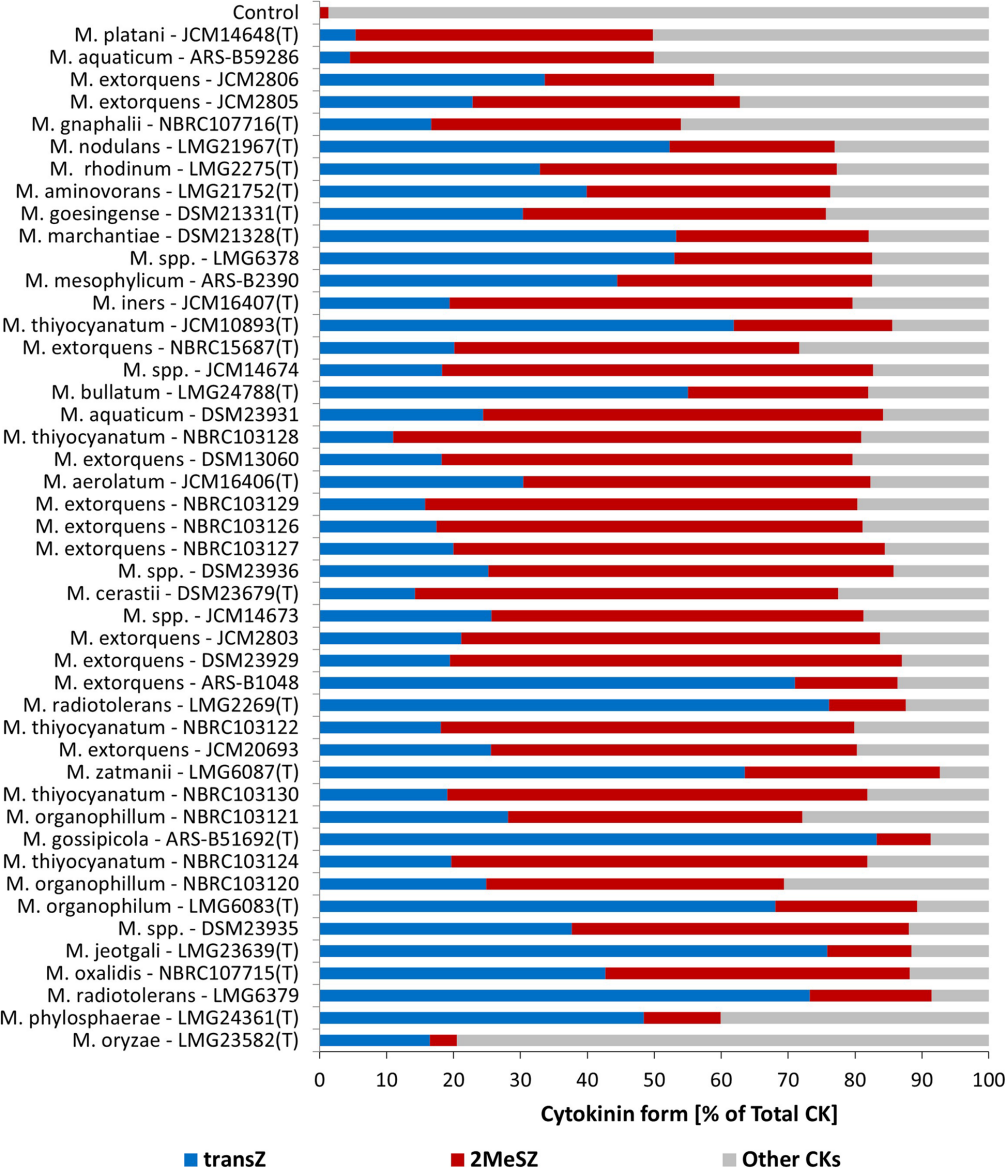
Share of trans-Zeatin (transZ), 2-methylthio-Zeatin (2MeSZ), and other cytokinin (CK) forms in 46 *Methylobacterium* strains. Strains were cultured in vitro in the DSM125 minimal medium until they reached the early stationary phase. Cytokinin were analysed in the cell-free bacteria supernatants using HPLC-(ESI +)MS/MS. Strains are ordered according to the increasing total CK production (*n* = 3)

Of the 28 endogenous CKs monitored, 16 CKs forms were detected at different concentrations (Table S1). The HPLC–MS/MS analysis of CK levels in bacteria supernatants consistently revealed substantial levels of the most active CK-FB forms (mainly tZ, and cis-Zeatin (cZ) in a lower number of strains), as well as relatively high levels of the 2-methylthio-Zeatin conjugate (2MeSZ). In most bacteria strains analyzed, tZ (0.45 – 82.16 pmol mL^−1^) and 2MeSZ (4.5 – 54.3 pmol mL^−1^) were the most abundant compounds, representing over 70% of the total detected CK content (Fig. 2).

**Fig. 3:**
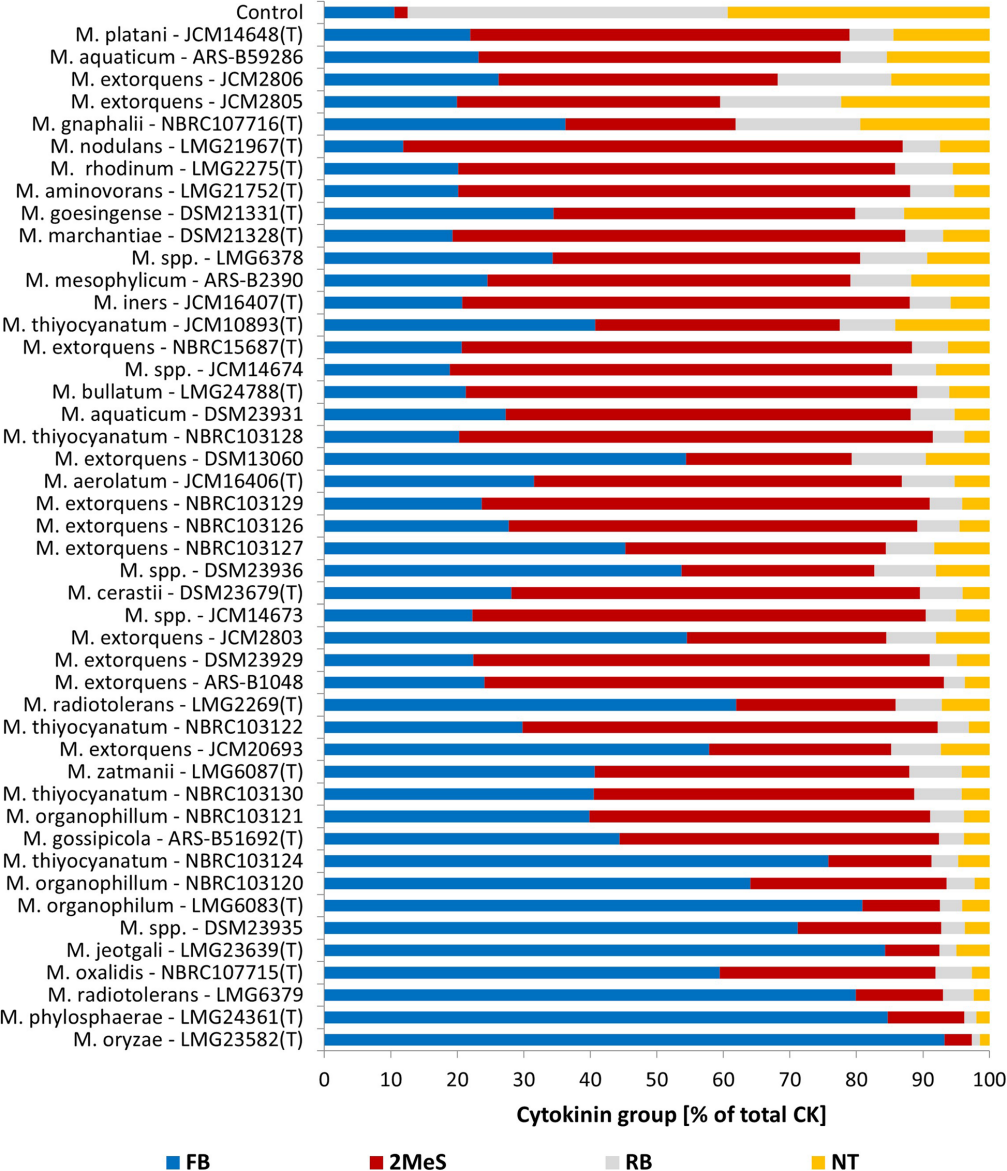
Share of free bases (FB), methylthiols (2-MeS), ribosides (RB), and nucleotides (NT) in 46 *Methylobacterium* strains. Strains were cultured in vitro in the DSM125 minimal medium until they reached the early stationary phase. Cytokinin were analysed in the cell-free bacteria supernatants using HPLC-(ESI +)MS/MS. Strains are ordered according to the increasing total CK production (*n* = 3)

For most of the analysed strains, the main CK group secreted to bacterial supernatants were the most biologically active free bases (CK-FB), followed by methylthiols (2MeS-CKs), followed by lower levels of ribosides (CK-RB) and nucleotides (CK-NT) (Fig. 3). No considerable differences were observed between the strains regarding the distribution of two most abundant CK groups; however, whenever the ability to produce CK-FBs was more pronounced among the strains, the total 2MeS-CK concentration was markedly decreased. The *M. oryzae* strain LMG23582(T), was the only case for which cZ was detected in higher quantity compared to tZ isomer (146.6 pmol mL^−1^ and 31.5 pmol mL^−1^, respectively).

Across the inventory of bacteria strains, CK-RB and CK-NT derivatives of isopentenyladenine (iP) were identified at higher concentrations (0.64–6.26 pmol mL^−1^ for isopentenyladenosine (iPR), and 1.41 – 4.61 pmol mL^−1^ for isopentenyladenine nucleotide (iPNT)), relative to the CK-RB and CK-NT derivatives of any of Dihydrozeatin (DHZ), tZ, or cZ, which were detectable at only minute levels in the supernatant of *Methylobacterium* strains. Likewise, other forms such as DHZ, 2-methylthio-Zeatin riboside (2MeSZR), and 2-methylthio-Isopentenyladenosine (2MeSiPR) were detected at very low levels. Glucoside derivatives and aromatic CKs were not present in the supernatants of any of the *Methylobacterium* strains.

**Fig. 4:**
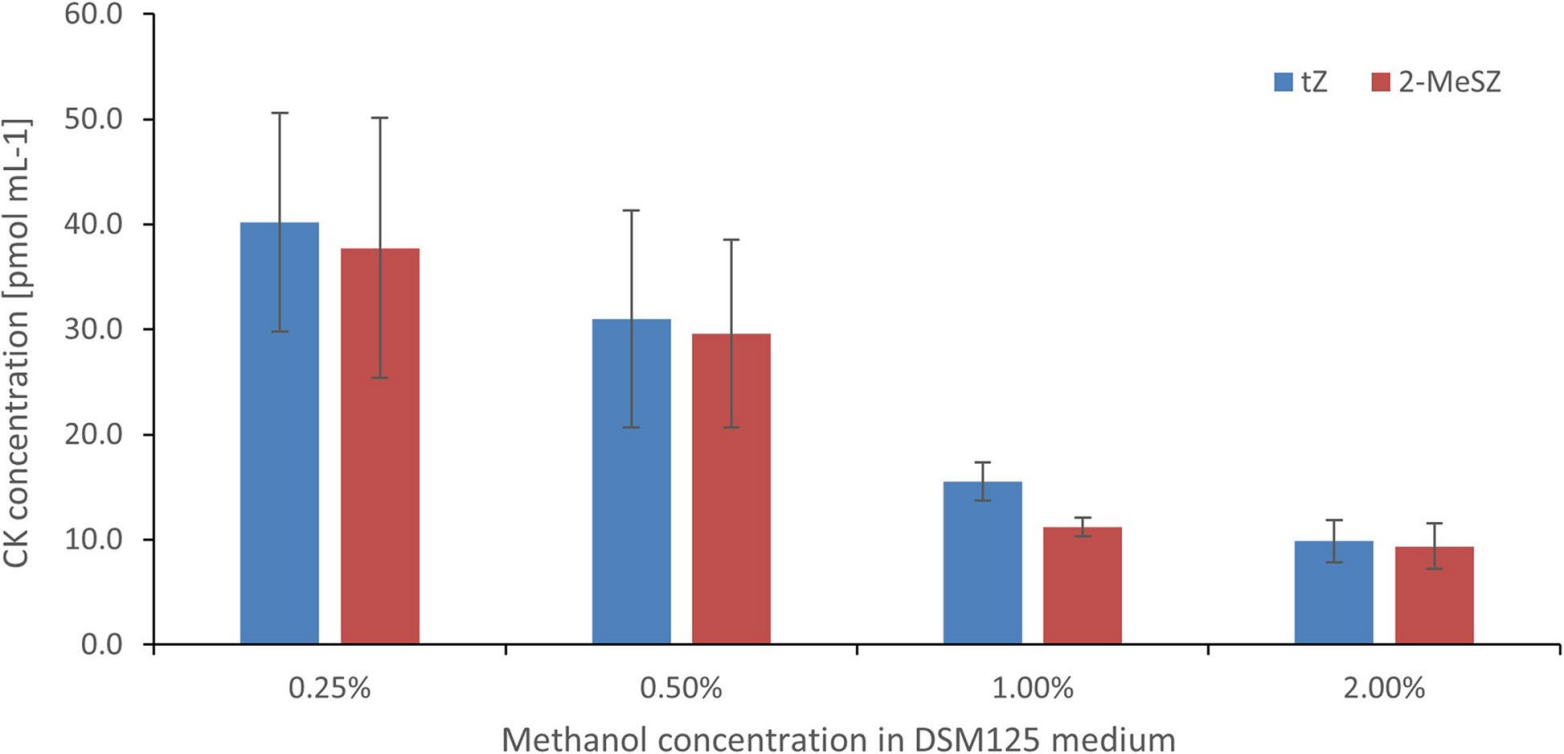
Cytokinin (CK) concentration (pmol mL^−1^) in *Methylobacterium oryzae* — LMG23582(T), cultured under different concentrations of methanol (0.25 – 2.00%). Strains were cultured in vitro in the DSM125 minimal medium until they reached the early stationary phase. Cytokinin were analysed in the cell-free bacteria supernatants using HPLC-(ESI +)MS/MS. Values are means ± SE of 3 replicates. (*n* = 3)

The analysis of CK levels in the supernatants of the most productive strain, *M. oryzae* LMG23582(T), cultured under different concentrations of CH_3_OH (0.25%, 0.5%, 1% and 2%) revealed a clear trend in CK secretion, whereby the hormone production was intensified by the decreasing concentration of CH_3_OH in the growth media (Fig. 4, Table S2).

No endogenous ABA was detected from any of the analysed strains.

### Indole-3-acetic acid (IAA) in *Methylobacterium* strains

**Fig. 5:**
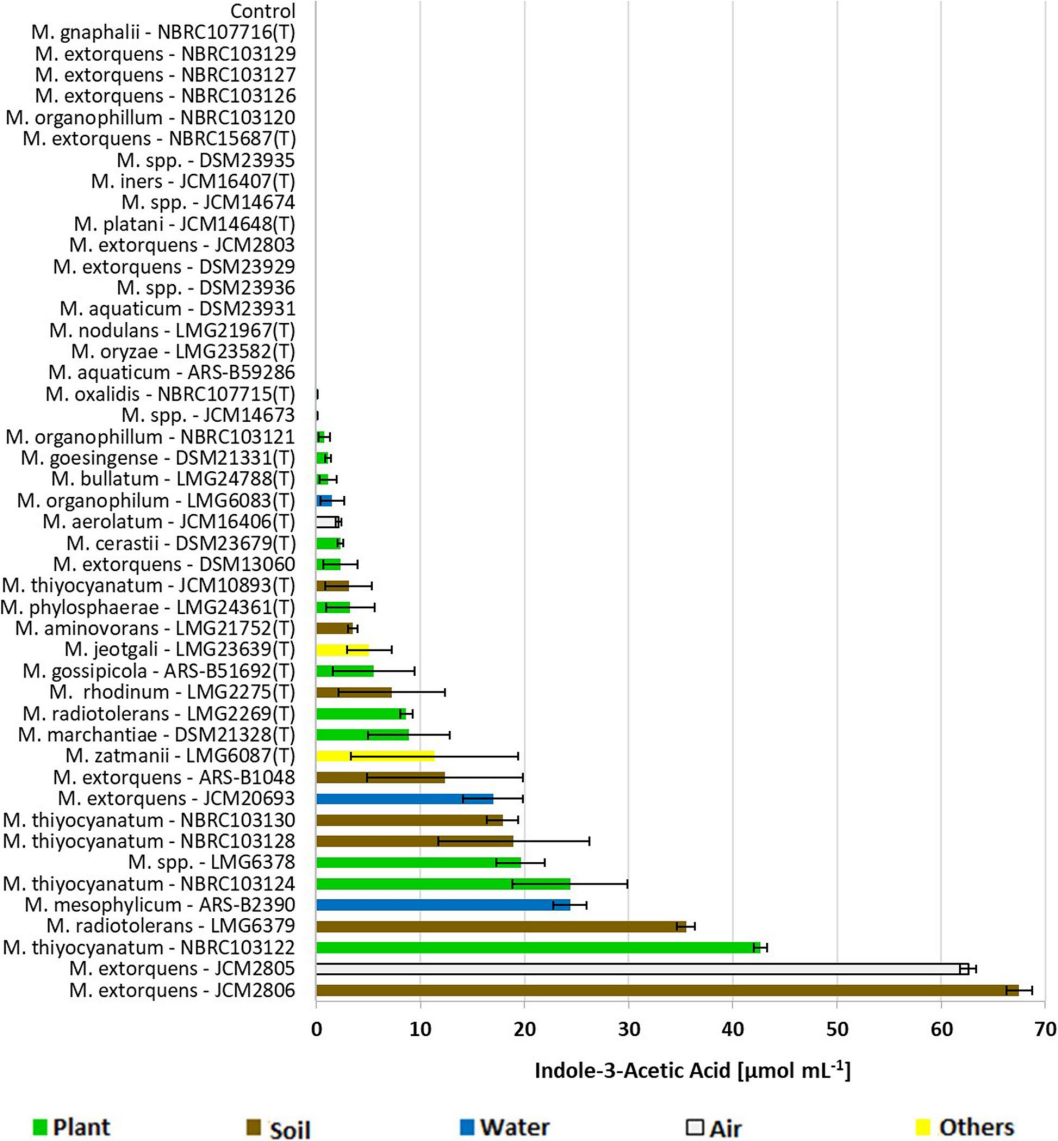
Indole-3-Acetic Acid concentration (IAA; µg mL^−1^) in 46 *Methylobacterium* strains. Strains were cultured in vitro in R2 broth supplemented with 2.5 mM L-tryptophan until they reached the early stationary phase. Indole-3-Acetic Acid concentration was measured spectrophotometrically using a colorimetric method. Values are means ± SE of 3 replicates

The colorimetric method used in this study to quantify the levels of bacterial IAA revealed that out of the 46 *Methylobacterium* strains, 29 were able to secrete IAA at varying concentrations (0.11 – 67.47 µmol mL^−1^) after incubation with an L-tryptophan precursor. Out of the 12 strains originally isolated from soil or rhizosphere, 8 secreted IAA in concentrations ranging from 3.14 to 67.47 µmol mL^−1^ (Fig. 5). Supernatants of two *M. extorquens* strains (JCM2805 and JCM2806) had the highest IAA production (over 60 µmol mL^−1^), which corresponded with one of the lowest total CK contents (below 20 pmol mL^−1^). By contrast, the most active CK producer, *M. oryzae* (LMG23582(T)) did not secrete detectable IAA levels to the culture supernatant, while another strain characterised by high CK concentration in the culture supernatants, *M. phylosphaerae* (LMG24361(T)) released only minute IAA levels (below 4 µmol mL^−1^). On the other hand, *M. thiyocyanatum* (NBRC103122) and *M. radiotolerans* (LMG6379) were associated with relatively high levels of both IAA conversion (over 35 µmol mL^−1^) and total CKs (over 70 pmol mL^−1^).

Generally, the levels of IAA converted in *Methylobacterium* cultures from L-tryptophan supplement, were significantly higher compared to the secreted CK levels. However, no endogenous IAA was detected in any cultures that were not supplemented with L-tryptophan, a direct precursor of IAA biosynthesis.

## Discussion

Pink pigmented facultative methylotrophs (PPFMs) belonging to the *Methylobacterium* genus are a unique group of plant-associated microorganisms, that are considerably beneficial to their hosts [9, 25, 34, 35, 38, 39]. They are ubiquitous in nature and reside in diverse environments such as leaf surfaces, plant tissues, soil, and in the air. Strains of *Methylobacterium* are known to enhance seed germination, seedling growth, and increase systemic resistance in multiple plant species [8, 31, 34, 40, 41] Many of these important plant growth-promoting bacteria (PGPB) have a natural capability of synthesizing high levels of phytohormones including CKs [6, 34, 42, 43]. Building upon the findings of our previous study [34] which looked at only 7 strains, we have now produced the most comprehensive inventory of CK production across a much wider breadth of *Methylobacterium* species with the most advanced, available technology that couples the precise separation methodology of high-performance liquid chromatography (HPLC) with tandem mass spectrometry (MS/MS). Our results confirm that all *Methylobacteria* can produce CKs, albeit at quite varying levels, which surpass de novo production of related hormones (e.g., ABA, IAA) even though they are demonstrably capable of making IAA. Additionally, our data indicate that the intensity of CK secretion *by Methylobacterium* depends on the availability of methanol in the environment.

The presence of CKs among strains of *Methylobacterium* have been reported to date (e.g., [34]); however, most of the earlier studies could only detect up to 4 CK forms at a time based on the dated hormone extraction techniques and detection limits [44–46]. Previously, a comprehensive hormone purification protocol enabled the screening of 25 CK compounds from rhizosphere bacteria with successful detection and identification of 11 forms among *Rhizobium* strains [47]. Using a similar approach, in addition to detecting 16 different CKs within *Methylobacterium*, our study illustrates significant variation in the inventory of CK production by different *Methylobacterium* strains — specifically, with respect to the diversity of CKs produced and to the secretion of the highly active tZ free base and its derivatives. Bacterial supernatants used for CK profiling were aliquoted from the 5-day old cultures, when *Methylobacteria* reached their early stationary phase. Detected levels of CKs often correspond closely to the biomass accumulation of the analysed isolates; moreover, such a relationship is a good indicator of the strain growth rate in their natural environment, and their ability to successfully colonise the inoculated plant tissues. The data presented here follow trends regarding the differences in CK profiles that are similar to those among the 7 *Methylobacterium* strains analysed in our previous study [34].

Studies involving plant-associated bacteria, including rhizobia, have previously documented the production of mainly cZ- and iP-types of CKs, while our investigation shows that *Methylobacteria* are most distinctly remarkable through their prominent secretion of the bioactive tZ form. Most widely known for its presence in higher plants, tZ, can bind to at least two plant CK receptors with high affinity and it was characterised as the most active CK [48]. In plant systems, tZ participates in growth and development [26, 49], regulation of plant immunity [50], or the alleviation of photoperiod stress [51]. Within the microbial community, previous evaluation of the CK profiles in mycelial biomass of medicinal mushrooms revealed tZ as the most abundant CK form in most species [52]; yet, the regulatory functions of tZ in mushroom growth could not be fully elucidated. By contrast, an examination of CK production by temperate forest soil fungi indicated the complete absence of tZ across all 20 strains investigated [53]. In another study involving yeasts from different taxonomic groups and habitats, Z-type CKs were detected at high levels; however, the differentiation between discrete Z isomers was not established, nor was a clear conclusion drawn regarding a possible role of Z synthesis in the growth of yeasts [54].

Comparison of phytohormone profiles of the 46 *Methylobacterium* strains revealed remarkable consistencies in the distribution and levels of CK groups and types, indicating there is a characteristic CK pattern among different species of this genus. In general, tZ was detected in higher abundance relative to cZ levels, with CK-FB forms found at higher concentrations than CK-NT and CK-RB forms. Though our work indicated greater presence of the highly active tZ among the representatives of *Methylobacterium* genus, its isomer, cZ, was also detectable.

Research is accumulating that suggests that cZ forms can have great agricultural potential; beyond being involved in plant growth regulation, cZ can also play a crucial role in intermediate responses to stress including infection and herbivory [55] and the two isomer systems together are thought to offer a dual level of response, as either hard-hitting and brief, or with low activity and longer duration [56]. trans-Zeatin was found at high levels in some important experimental plants (i.e., Arabidopsis), while in legumes like chickpea and field pea, cZ was identified as the predominant CK form [57, 58]. Diversity in the relative abundance of CK types across different plant species is also an attribute mirrored in the CK profiles of the examined *Methylobacterium*. It is possible that selecting a bacterial strain with a CK production profile similar to that of the target plant could work to enhance the effects of beneficial phytohormones in the host through an additive mechanism. Conversely though, selecting a strain that has a CK profile opposite but complementary to the host plant, may in fact work to achieve positive outcomes through supplementation of hormones which have low endogenous expression with longer duration. Such beneficial effects may manifest as improvements in response, and tolerance, to biotic and abiotic stresses. This may form the basis for the next chapter of research at the biochemical interface between PGPB and their hosts.

Methylthiol-CKs (2MeS-CKs) were the second most abundant fraction in *Methylobacterium* CK profiles. Methylthiol-CKs are the least characterized CK group as the breadth of their physiological effects and participation in signalling cascades have yet to be even partially elucidated [28]. This CK group is often suggested to be mainly of microbial origin, and a modification of the tRNA degradation pathway was proposed as a possible route for 2MeS-CK biosynthesis [28, 59] including in *Methylobacterium* [29]. High levels of 2MeS-CKs were detected in other studies involving bacterial symbionts such as *Rhizobia* [44, 45, 47]. Consequently, our findings align with earlier work by establishing a relatively high production rate of 2MeS-CKs among bacteria strains and consistency of the inverse relationship previously observed between 2MeS-CK production and that of known active forms such as tZ [28, 47].

Riboside (CK-RB) and nucleotide (CK-NT) forms are usually considered to be less biologically active, as CK-NTs are the stable precursors to more active CK forms [26, 60], and CK-RBs are regarded as more suitable for transport within plants [49, 61]. On the other hand, it has been proposed that CK-RBs might have roles beyond transport, including potential direct activity [62]. Both the CK-NT and CK-RB forms were detected, in much lower abundance than CK-FBs, across the analyzed *Methylobacterium* strains. As the less bioactive precursors, CK-NTs are more likely retained inside the bacterial cells while their secretion to the supernatant is not expected. Overall, only NT and RB derivatives of iP were detected at higher levels relative to other NT and RB forms evaluated. The isoprenoid side chain of isopentenyladenine is synthesised via the isopentenyl-dependent mevalonate pathway in microbes (MVA) [63] and, thus, the presence of iP-type CKs suggests this pathway exists in *Methylobacterium*. Yet, the concentrations of iP in *Methylobacterium* supernatants were several times lower than those of tZ, perhaps because iP is less a stable product of the pathway and can be quickly hydroxylated to tZ by cytochrome P_450_ monooxygenases [26], or because of the dominant tZ production via tRNA modification [29].

Glucoside-CK conjugates were not detected in any of *Methylobacterium* strains, and this is consistent with reported evolutionary patterns of CKs in prokaryotic organisms compared to higher plants in which glucosylation is exclusively found [47, 64].

One of the advantages of *Methylobacteria* over many other plant-associated microbes is their ability to use single carbon compounds, such as methanol, as their sole carbon source. It was hypothesised that *Methylobacterium* strains release CKs to stimulate plant cell division that results in the production of methanol, the by-product of cell wall metabolism [24, 25]. This characteristic, unique to methylotrophic microbes, is particularly beneficial under natural growth conditions, often characterized by the limited availability of energy-rich molecules like soluble carbohydrates. In our study, the increasing concentration of methanol in the medium (from 0.25% to 2%) caused a decrease in the levels of tZ and 2-MeSZ in the supernatant of *M. oryzae* (LMG23582(T)) from 40.2 and 37.74 pmol mL^−1^ to 9.88 and 9.38 pmol mL^−1^, respectively. These results further indicate that bacterial CK production is stimulated by the reduced availability of methanol in the growth environment end explain the potential benefits from the plant-bacteria interaction to the *Methylobacterium* partner.

Indole-3-acetic acid (IAA) production from L-tryptophan by *Methylobacterium* strains was detected through use of the Salkowski reagent and quantified using a common spectrophotometric technique for measuring IAA production of microbial origin [65–67]. Without L-tryptophan supplementation no endogenous IAA was detected; however, with L-tryptophan, the total IAA concentration varied from 0 to 67.47 µmol mL^−1^. Among a range of biological functions that IAA participates in, is a stimulation of plant root growth [68]. Earlier studies with microorganisms showed IAA producers are often, like many *Methylobacteria*, gram-negative microbes [69]. In gram-negative rhizobacterial isolates from *Coleus* rhizosphere, IAA production was found at concentrations between 1,312 and 1,484 µmol mL^−1^ using spectrophotometric methods [70]. Certain obligate and facultative methylotrophic bacteria, in the presence of L-tryptophan, were reported to produce IAA in the range of 28 – 80 µmol mL^−1^ [65] which is comparable to the findings of this study.

In our investigation, out of 12 *Methylobacterium* strains of soil origin, 8 were found to have measurable levels of IAA when fed with L-tryptophan. While not all 12 soil-borne *Methylobacteria* could produce detectable levels of IAA, the biosynthesis of this compound does appear to be linked to the strains isolated from the phytobiome (leaves, roots) compared to those without a link to a plant (air, water, other sources). Furthermore, although the inverse relationship between CK production and IAA conversion rate was not obvious for all the analysed *Methylobacteria*, the strains capable of secreting the most abundant CK levels (i.e., *M. oryzae* (LMG23582(T)) or *M. phylosphaerae* ((LMG24361(T)) were not converting L-tryptophan to IAA effectively. Moreover, the high IAA producers were characterised by low CK concentrations in their culture supernatants. This may indicate specialization and adaptation of *Methylobacterium* strains for their respective ecological niches.

## Conclusions

Herein, we provide the most comprehensive survey of CK production among many strains of the *Methylobacterium* genus. We have established that *Methylobacterium* species are able to secrete a wide range of the most active CK form, tZ, and this occurred at levels higher than any other PGPB, which could indeed benefit plant hosts. Our data indicate that a detailed bacterial CK profile can aid in the selection of *Methylobacterium* candidates that are highly effective as plant growth promoters. Retrospectively, the findings of earlier investigations [33, 34, 71, 72] align with the results presented herein, whereby the *M. oryzae* (LMG23582(T)) strain is likely mediating its marked growth-promoting effects uniquely through its high CK production. The knowledge of CK production could help reduce research costs associated with laboratory consumables, equipment, and labour associated with large scale greenhouse or field-trial experiments by streamlining the process of identifying the most potentially effective strains for bioinoculant development. In the future, other high tZ-producing strains of *Methylobacterium* genus should be investigated for growth-promoting and stress-alleviating effects in controlled experiments to further elucidate their role in plant growth and crop protection.

Testing the effect of the methanol concentration in the growth medium, supported the hypothesis that *Methylobacteria* secrete CKs to stimulate plant cell division and release of methanol that microbial symbionts can subsequently utilize as a carbon source. By contrast, other hormones scanned for (ABA, IAA) were not endogenously detected, unless a precursor was fed to the cells (IAA). In the latter case, IAA conversion capacity from L-tryptophan was inversely related to CK levels among many of the analysed strains.

## Methods

### Bacterial strains and growth conditions

**Fig. 6:**
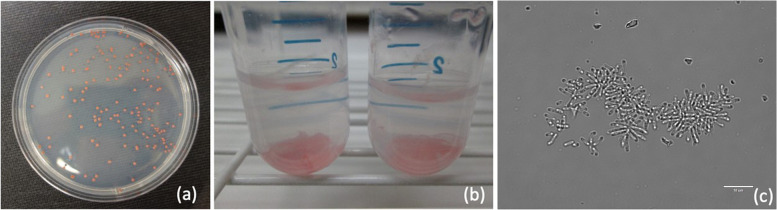
*Methylobacterium organophilum* growth on DSM125 agar plate (**a**) and in DSM125 liquid medium (**b**). Microscopic image of aggregate formation by *Methylobacterium organophilum* cells in liquid cultures (**c**)

Freeze-dried cultures of 46 *Methylobacterium* spp. strains were obtained from five microbe collections: the Agricultural Research Service (ARS) of the Northern Regional Research Laboratory (NRRL), the Belgian Coordinated Collections of Microorganisms (BCCM/LMG), the Deutsche Sammlung von Mikroorganismen und Zellkulturen (DSMZ) [“German Collection of Microorganisms and Cell Cultures”], the Japan Collection of Microorganisms (JCM), and the National Institute of Technology and Evaluation’s (NITE) Biological Resource Center (NBRC). The strain selection was divided as follows; ARS (NRRL) – United States of America (*n* = 5), BCCM/LMG—Belgium (*n* = 12), DSMZ – Germany (*n* = 8), JCM – Japan (*n* = 10), and NITE (NBRC) – Japan (*n* = 12). The strains were originally isolated from different biological sources including plant organs (phyllosphere, flowers, roots, moss tissue), soil, water, air, and other materials. The detailed information on strain taxonomy, origin, known characteristics and applications is provided in Table 1. The freeze-dried strains were revived in nutrient-rich R2 broth (VWR, Mississauga, Canada) and maintained as 15% (v/v) glycerol stocks at -80 °C. The analysed strains varied in their growth characteristics, such as the intensity of pellet pigmentation (white, orange, pale pink to intense pink), growth rate (5 mg – 50 mg pellet in 15 ml culture), or ability to aggregation and biofilm formation (Fig. 6).

### Screening for bacterial phytohormone production

For phytohormone analysis, *Methylobacterium* stocks were aseptically streaked onto agar plates and liquid cultures were started by suspending the bacterial single colonies with a sterile disposable loop in 50 mL Falcon tubes containing 15 mL of selective minimal medium prepared according to the DSMZ *Methylobacterium* medium recipe (index #125): KNO_3_—1.00 g; MgSO_4_ × 7H_2_O—0.2 g; CaCl_2_ × 2H_2_O—0.02 g; Na_2_HPO_4_—0.23 g; NaH_2_PO_4_—0.07 g; FeSO_4_ × 7H_2_O—1.00 mg; CuSO_4_ × 5H_2_O – 5 μg; H_3_BO_3_—10 μg; MnSO_4_ × 5H_2_O – 10 μg; ZnSO_4_ × 7H_2_O—70 μg; MoO_3_ – 10 μg; H_2_O—1000 ml; CH_3_OH—5 ml; pH 6.8). Aseptic bacteria cultures were incubated in a rotary shaker (28 °C/250 rpm). Supernatant samples were harvested on 5–7 day of the culture, when *Methylobacterium* reached the late exponential/early stationary phase (OD_600_ = 0.6–1.2, depending on the strain). To determine cell concentration in the liquid culture, bacterial growth was monitored spectrophotometrically by measuring OD_600_ and confirmed by cell count using a standard serial dilution technique.

To find out if the level of methanol in the growth medium affects the rate of CK production by *Methylobacterium*, the independent batch of *M. oryzae* strain LMG23582(T) was cultured in the 125DSM media supplemented with increasing concentrations of CH_3_OH (0.25%, 0.5%, 1% and 2% v/v). All other experimental conditions remained the same as described above.

### Purification of cytokinins (CKs) and abscisic acid (ABA)

A modified protocol for liquid culture medium was used for CK and ABA extraction and quantification [47]. The levels of bacterial phytohormones were measured in 12 mL of the cell-free supernatant obtained after centrifugation (Thermo Scientific, Sorvall ST16; 10 min, 4,696 × g) of harvested in vitro cultures. Samples of culture medium that were not inoculated with *Methylobacterium* were subjected to analysis as a negative control, to determine that the CKs detected in the bacteria culture supernatant were exclusively of microbial origin.

The profiles of 28 CK metabolites and ABA were analyzed, including: CK free bases (CK-FBs), their riboside (CK-RBs) and CK-nucleotide (CK-NTs) derivatives, CK-glucoside (CK-GLUCs) and methylthiol conjugates (2MeS-CKs), and select aromatic CKs. The cell-free supernatant samples were mixed with 1 ml of cold (–20 °C) modified Bieleski #2 solvent (CH_3_OH:H_2_O:HCO_2_H [15:4:1, vol/vol/vol]) using vortex. Internal standards were added to each sample to enable endogenous hormone quantification through the isotope dilution method. Samples were spiked with 144.7 ng of ^2^H_4_-ABA (NRC-PBI, Saskatchewan, SK, Canada) and 10 ng of each of the deuterated internal standard CKs (Table 2) obtained from OlChemIm Ltd. (Olomouc, Czech Republic). As deuterated standards of cis-Zeatin-type CKs were not commercially available at the time of this investigation, the levels of cis-compounds were quantified based on the recovery of the deuterated standards of the corresponding trans-compounds.

**Table 2:** Cytokinins (CKs) scanned for using liquid chromatography-positive electrospray ionization tandem mass spectrometry in *Methylobacterium* supernatants

Cytokinin (CK)	Labelled CK Standard
**Nucleotides (mono- di- and triphosphates; CK-NTs)**	
trans-Zeatin nucleotide (tZNT)	[^2^H_3_]DHZRMP
cis-Zeatin nucleotide (cZNT)	
Dihydrozeatin nucleotide (DHZNT)	
Isopentenyladenine nucleotide (iPNT)	[^2^H_6_]iPRMP
**Ribosides (CK-RBs)**	
trans-Zeatin riboside (tZR)	[^2^H_5_]ZR
cis-Zeatin riboside (cZR)	
Dihydrozeatin riboside (DHZR)	[^2^H_3_]DHZR
Isopentenyladenosine (iPR)	[^2^H_6_]iPR
**Free Bases (CK-FBs)**	
trans-Zeatin (tZ)	[^2^H_5_]Z
cis-Zeatin (cZ)	
Dihydrozeatin (DHZ)	[^2^H_3_]DHZ
Isopentenyladenine (iP)	[^2^H_6_]iP
**Glucosides (CK-GLUCs)**	
trans-Zeatin-O-glucoside (tZOG)	[^2^H_5_]ZOG
cis-Zeatin-O-glucoside (cZOG)	
Dihydrozeatin-O-glucoside (DHZOG)	[^2^H_7_]DHZOG
trans-Zeatin-O-glucoside riboside (tZROG)	[^2^H_5_]ZROG
cis-Zeatin-O-glucoside riboside (cZROG)	
Dihydrozeatin-O-glucoside riboside (DHZROG)	[^2^H_7_]DHZROG
trans-Zeatin-9-glucoside (tZ9G)	[^2^H_5_]Z9G
cis-Zeatin-9-glucoside (cZ9G)	
Dihydrozeatin-9-glucoside (DHZ9G)	[^2^H_5_]DHZ9G
**Methylthiols (2MeS-CKs)**	
2-Methylthio-Zeatin (2MeSZ)^a^	[^2^H_5_]2MeStZ
2-Methylthio-Zeatin riboside (2MeSZR)^a^	[^2^H_5_]2MeStZR
2-Methylthio-N^6^-isopentenyladenine (2MeSiP)	[^2^H_6_]2MeSiP
2-Methylthio-N^6^-isopentenyladenosine (2MeSiPR)	[^2^H_6_]2MeSiPR

^a^The analytical procedure used in this study does not facilitate separation of 2MeStZ and 2MeStZR from their corresponding cis-isomers, 2MeScZ and 2MeScZR. Therefore, the levels of the methylthiolated Z-type CKs were reported as total 2MeSZ and total 2MeSZR

Samples were incubated overnight at –20 °C and evaporated to dryness at 35 °C under vacuum (Model SPD111V; Thermo Scientific, Ottawa, Canada). The residues were reconstituted in 200 μl of 1 M HCO_2_H (pH 1.4) for purification and concentration of metabolites using automated Gilson SPE 215 Solid Phase Extraction System (Gilson Inc., Middleton, WI, USA). The hormone-containing fraction was purified and concentrated on a mixed mode, reverse-phase, cation-exchange SPE cartridges (Oasis MCX, Waters, Mississauga, Canada) as previously described [73], with modifications [74]. Cartridges were activated with high-performance liquid chromatography (HPLC) grade CH_3_OH and were equilibrated using 1 M HCO_2_H (pH 1.4). After equilibration, each sample was loaded and washed with 1 M HCO_2_H (pH 1.4). Hormone groups were eluted based on the charge and hydrophobicity level. Abscisic acid was eluted first using CH_3_OH. Cytokinin nucleotides (CK-NTs) were eluted next with 0.35 M NH_4_OH. Cytokinin free bases, CK-RBs, 2MeS-CKs, CK-GLUCs and aromatic CKs were retained on the column based on charge and hydrophobic properties and, therefore, were lastly eluted, using 0.35 M NH_4_OH in 60% [vol/vol] CH_3_OH. Each collected elution was evaporated to dryness at 35 °C under vacuum.

Since intact CK-NTs cannot be analyzed directly with the present method, they were dephosphorylated by overnight incubation with 3 units of calf-intestinal alkaline phosphatase (New England Biolabs Ltd., Whitby, ON, Canada) in 1 mL of 0.1 M ethanolamine-HCl (pH 10.4) at 37 °C to form CK-RBs. This detection method of CK-NTs potentially reflects pooled contribution of mono, di- or triphosphates in that the isopentenyl or hydroxylated moiety may be transferred to AMP, ADP or ATP [58]. The resulting CK-RBs were evaporated to dryness at 35 °C under vacuum. The samples were reconstituted in double-distilled water (dd-H_2_O) and were further isolated on a reversed-phase C18 solid phase extraction cartridges (C18/14%, Canadian Life Science, Peterborough, ON, Canada), which were first activated with CH_3_OH and equilibrated using dd-H_2_O. Samples containing CK-RBs were loaded onto the C18 cartridges and were allowed to pass through the column by gravity. The sorbent was washed with dd-H_2_O. Resultant CK-RBs were eluted using 100% CH_3_OH and eluents were evaporated to dryness at 35 °C under vacuum.

Prior to LC–MS/MS analysis, all phytohormone samples were dissolved in starting conditions solvent (CH_3_OH:CH_3_CO_2_H:dd-H_2_O [5:0.08:94.92, vol/vol/vol] for ABA and CH_3_CN:CH_3_CO_2_H:dd-H_2_O [5:0.08:94.92, vol/vol/vol] for CKs) and were centrifuged at 12,320 × *g* for 10 min to remove any solid particles (Thermo Scientific). The supernatants were stored at –20 °C until further processing.

### High performance liquid chromatography—electrospray ionization—tandem mass spectrometry (HPLC-(ESI)-MS/MS) analysis

Levels of plant growth regulators were measured using high performance liquid chromatography-electrospray ionization-tandem mass spectrometry (HPLC-(ESI)-MS/MS; QTrap5500; ABI Sciex Concord Ontario, Canada, coupled with Agilent 1100 series HPLC; Agilent, Mississauga, ON, Canada). An aliquot (40 µl) was injected on a Luna C18 reversed-phase column (3 μm, 150 × 2.0 mm; Phenomenex, Torrance, Canada). Abscisic acid was eluted using component A: H_2_O with 0.08% CH_3_CO_2_H and component B: CH_3_OH, both with 0.08% CH_3_CO_2_H, at a flow rate of 0.2 mL min^−1^. Cytokinins were eluted with an increasing gradient of 0.08% CH_3_CO_2_H in CH_3_CN (A) mixed with 0.08% CH_3_CO_2_H in ddH_2_O (B), at a flow rate of 0.2 ml min^–1^. For the ABA, the initial conditions of 50% B remained constant for 4 min, then changed linearly to 95% B over 6 min. This ratio was held constant for 1 min before immediately returning to starting conditions and re-equilibrating for 20 min. The initial conditions for CK groups were 5% A and 95% B, changing linearly in 17 min to 95% A and 5% B. Conditions remained constant for 5 min, and then, immediately returned to initial conditions for 18 min of re-equilibration.

Abscisic acid was analyzed in negative ionization mode and CKs were analyzed in positive ionization mode. Phytohormones were identified based on their analyte specific retention times and multiple reaction monitoring (MRM) channels. All data were analyzed with Analyst 1.6.2 software (AB SCIEX, Concord, ON, Canada). Hormone concentrations were established according to isotope dilution analysis upon direct comparison of the endogenous analyte peak area against the recovered internal standard [74]. Quantification of the cis isomers of zeatin-type CKs (cZNT, cZR, cZ, cZOG, cZ9G, cZROG) was performed relative to the recovery of labeled tZ-types and retention time of unlabeled cZ standards. Because using C18 type HPLC column does not facilitate separation of 2MeStZ and 2MeStZR from their cis-isomers [75], the levels of methylthiolated Z-type CKs were reported as total 2MeSZ and 2MeSZR.

### Indole-3-acetic acid (IAA)

For the evaluation of the IAA production capacity, 46 strains were grown in the dark in R2 broth supplemented with L-tryptophan (2.5 mM) and in L-tryptophan-free medium. Seven-days-old cultures that were in late exponential phase, were subjected to quantitative screening for IAA production via colorimetric method using Salkowski’s reagent [76]. Obtained by centrifugation (10 min, 4,696 × g), cell-free suspensions of each strain (1 vol) were incubated for 30 min (RT) with Salkowski reagent (1 ml 0.5 M FeCl_3_ in 50 ml of 35% HClO_4_) (0.5 vol). Indole-3-acetic acid concentration was quantified spectrophotometrically at wavelength 540 nm. The spectrophotometer was calibrated against a blank media with L-tryptophan mixed with the Salkowski reagent. To determine the sensitivity and the operating range of IAA concentrations in bacterial cultures, a calibration curve was calculated based on dilution series of authentic IAA (0–570 µmol mL^−1^) (Sigma-Aldrich, Inc., St. Louis, MO, USA).

### Statistical analysis

The experiments evaluating CKs, ABA, and IAA production by *Methylobacterium* were performed in three biological replicates (independent cultures) for each of the tested strains. The levels of bacterial phytohormones were expressed as per pmol or µmol in ml of the culture supernatant (*n* = 3 ± SE). Blank media samples (control) were processed and analysed using the same methodologies to account for any background levels of phytohormones.

## Supplementary Information

**Additional file 1: Table S1.** Cytokinin (CK) concentrations (pmol mL-1) detected by HPLC-(ESI+)MS/MS in the cell-free supernatants of 46 *Methylobacterium* strains cultured in vitro in the DSM125 minimal medium. CK production was tested in the early stationary phase (5 – 7 days). Bacterial CKs scanned for by HPLC-(ESI+)MS/MS include: free base, riboside and nucleotide forms of Dihydrozeatin (DZ), trans-Zeatin (tZ) cis-Zeatin (cZ), and Isopentenyladenine (iP), and 2-methylthio-Zeatin (2MeSZ), 2-methylthio-Isopentenyladenine (2MeSiP), 2-methylthio-Zeatin riboside (2MeSZR), 2-methylthio-Isopentenyladenosine (MeSiPR). Values are means ± SE of 3 replicates; n.d. – not detected. **Table S2.** Cytokinin (CK) concentrations (pmol mL-1) detected by HPLC-(ESI+)MS/MS in the cell-free supernatants of *Methylobacterium oryzae* - LMG23582(T) strains cultured in vitro in the DSM125 minimal medium suplemented with various concentration of methanol. CK production was tested in 5-day old cultures. Bacterial CKs scanned for by HPLC-(ESI+)MS/MS include: free base, riboside and nucleotide forms of Dihydrozeatin (DZ), trans-Zeatin (tZ) cis-Zeatin (cZ), and Isopentenyladenine (iP), and 2-methylthio-Zeatin (2MeSZ), 2-methylthio-Isopentenyladenine (2MeSiP), 2-methylthio-Zeatin riboside (2MeSZR), 2-methylthio-Isopentenyladenosine (MeSiPR). Values are means ± SE of 3 replicates; n.d. – not detected.

## Data Availability

All data generated or analysed during this study are included in this published article and its supplementary information files.
